# Age Prediction with Resting‐State EEG: An Explainable Hybrid Deep Learning Framework Using Periodic and Aperiodic Features Across Eyes‐Open and Eyes‐Closed Conditions

**DOI:** 10.1002/alz70856_106423

**Published:** 2026-01-08

**Authors:** Hamed Azami, Ahmad Zandbagleh

**Affiliations:** ^1^ Centre for Addiction and Mental Health, Toronto, ON, Canada; ^2^ University of Toronto, Toronto, ON, Canada; ^3^ Toronto Dementia Research Alliance, Toronto, ON, Canada; ^4^ Iran University of Science and Technology, Tehran, Tehran, Iran (Islamic Republic of)

## Abstract

**Background:**

Resting‐state EEG (rsEEG) elucidates neural aging, yet many deep learning approaches rely on full‐spectrum features, focus on eyes‐closed conditions, and lack interpretability. Unlike power spectrum, periodic and aperiodic power spectral density (PAPSD) isolates oscillatory and background components, capturing subtle neural dynamics. Combining eyes‐open and eyes‐closed rsEEG may further improve performance by leveraging the distinct neural states each condition captures. We developed a hybrid deep learning framework integrating PAPSD across both conditions, employing LIME‐based explainable AI and data augmentation in a single approach to enhance clinical relevance and ensure robust generalizability.

**Methods:**

We used rsEEG data from 608 healthy participants (376 females) aged 20–70 years in the Dortmund Vital Study, recorded under both eyes‐open (three minutes) and eyes‐closed (three minutes) conditions using a 64‐channel system. Data were preprocessed with the HAPPE pipeline for artifact removal and filtered at 1–45 Hz. Our hybrid model combined convolutional neural network for spatial feature extraction, bidirectional long short‐term memory layers for inter‐frequency dependencies, and an attention mechanism to prioritize key features. Data augmentation (weighted sample combinations, Gaussian noise) enhanced robustness. The model was trained using a 10‐fold cross‐validation approach with Huber loss and the RMSprop optimizer, applying a regression paradigm to predict participant age and evaluating performance via mean absolute error (MAE).

**Results:**

The combined PAPSD model achieved a MAE of 2.24±0.22 years (R^2^=0.91±0.02), surpassing eyes‐closed (MAE: 2.92±0.10; R^2^=0.87±0.01) and eyes‐open (MAE: 4.14±0.22; R^2^=0.79±0.02) alone. Full‐spectrum power (eyes‐open + eyes‐closed) performed worse (MAE: 4.77±0.21; R^2^=0.75±0.01). LIME‐based insights highlighted the central region and beta frequency band as pivotal to age‐related neural changes.

**Conclusions:**

By integrating PAPSD features, eyes‐open and eyes‐closed recordings, data augmentation, and LIME‐based explainability, this framework offers robust, interpretable rsEEG‐based age prediction. These advances boost predictive accuracy and shed light on neural mechanisms of aging, informing future research in neurodevelopment and beyond, including Alzheimer's dementia.

